# Prevalence of anti-SARS-CoV-2 antibodies among blood donors in Northern Cape, KwaZulu-Natal, Eastern Cape, and Free State provinces of South Africa in January 2021.

**DOI:** 10.21203/rs.3.rs-233375/v1

**Published:** 2021-02-12

**Authors:** Wendy Sykes, Laurette Mhlanga, Ronel Swanevelder, Tanya Nadia Glatt, Eduard Grebe, Charl Coleman, Nadia Pieterson, Russel Cable, Alex Welte, Karin van den Berg, Marion Vermeulen

**Affiliations:** 1) South African National Blood Service,; 2) DSI-NRF Centre of Excellence in Epidemiological Modelling and Analysis (SACEMA), Stellenbosch University; 3) Western Cape Blood Service; 4) Vitalant Research Institute; 5) University of California San Francisco; 6) University of Cape Town; 7) University of the Free State

## Abstract

**Background:**

Population-level estimates of prevalence of anti-SARS-CoV-2 antibody positivity (seroprevalence) is a crucial epidemiological indicator for tracking the Covid-19 epidemic. Such data are in short supply, both internationally and in South Africa. The South African blood services (the South African National Blood Service, SANBS and the Western Cape Blood Service, WCBS) are coordinating a nationally representative survey of blood donors, which it is hoped can become a cost-effective surveillance method with validity for community-level seroprevalence estimation.

**Methods:**

Leveraging existing arrangements, SANBS human research ethics committee permission was obtained to test blood donations collected on predefined days (7^th^, 10^th^, 12^th^, 15^th^, 20^th^, 23^th^ and 25^th^ January) for anti-SARS-CoV-2 antibodies, using the Roche Elecsys Anti-SARS-CoV-2 assay on the cobas e411 platform currently available in the blood services’ donation testing laboratories. Using standard methods, prevalence analysis was done by province, age and race, allowing age to be regarded as either a continuous or categorical variable. Testing was performed in the Eastern Cape (EC), Free State (FS), KwaZulu Natal (ZN) and Northern Cape (NC) provinces.

**Results:**

We report on data from 4858 donors - 1457 in EC; 463 in NC; 831 in FS and 2107 in ZN. Prevalence varied substantially across race groups and between provinces, with seroprevalence among Black donors consistently several times higher than among White donors, and the other main population groups (Coloured and Asian) not consistently represented in all provinces. There is no clear evidence that seroprevalence among donors varies by age. Weighted net estimates of prevalence (in the core age range 15–69) by province *(compared with official clinically-confirmed COVID-19 case rates in mid-January 2021)* are: EC-63%*(2.8%)*, NC-32%*(2.2%)*, FS-46%*(2.4%)*, and ZN-52%*(2.4%)*.

**Conclusions:**

Our study demonstrates substantial differences in dissemination of SARS-CoV-2 infection between different race groups, most likely explained by historically based differences in socio-economic status and housing conditions. As has been seen in other areas, even such high seroprevalence does not guarantee population-level immunity against new outbreaks – probably due to viral evolution and waning of antibody neutralization. Despite its limitations, notably a ‘healthy donor’ effect, it seems plausible that these estimates are reasonably generalisable to actual population level anti-SARS-CoV-2 seroprevalence, but should be further verified.

## Introduction

Owing to the extraordinary public interest in all meaningful information about the COVID-19 pandemic, we here report on an initial analysis of an ongoing study. A fuller analysis will be described in the near future.

Given the substantial uncertainties around the true counts of cases of SARS-CoV-2 infection, and prior studies indicating that in many settings the confirmed case count is a small proportion of all laboratory confirmed infections, it is of ongoing importance to obtain credible estimates of *the prevalence of anti-SARS-CoV-2 antibody positivity* (seroprevalence), at the community level (1,2).

The prime objective of our study is to estimate seroprevalence in South African blood donors. Beyond that, we will pursue additional analyses such as 1) investigating how representative this prevalence is likely to be of community-level prevalence, 2) gathering data about the spectrum of disease associated with SARS-CoV-2 infection, and 3) considering options for leveraging ongoing surveillance of prevalence into real-time incidence estimates. In the present work, we focus on early seroprevalence estimates.

## Methods

The South African National Blood Service (SANBS – serving 8 of 9 provinces in South Africa) and Western Cape Blood Service (WCBS – servicing the Western Cape) obtained ethics clearance from the SANBS Human Research Ethics Committee to perform a SARS-CoV-2 seroprevalence study among South African blood donors. The protocol allowed for the testing of routinely collected donor screening samples on predefined ‘collection days’ (7^th^, 10^th^, 12^th^, 15^th^, 20^th^, 23^th^ and 25^th^ January); which were announced to blood centre staff, but without prior notice to potential donors. All donors underwent routine screening through a self-administered questionnaire, one-on-one assessment and a mini-health screening by blood centre staff. Donors who did not meet the routine donor eligibility criteria were excluded from donation and therefore from the study. To date, testing has been performed in the Eastern Cape (EC), Free State (FS), KwaZulu Natal (ZN) and Northern Cape (NC) provinces.

Samples collected at the time of donation, were tested for anti-SARS-CoV-2 antibodies, using the Roche Elecsys Anti-SARS-CoV-2 total immunoglobulin nucleocapsid assay on the cobas e411 platform already in use in the blood services. This assay, according to the package insert, has diagnostic specificity in excess of 99.5%, and near perfect sensitivity (point estimate of 100%) at 16 days post PCR positivity. We do not here explore various interesting nuances of how to define and estimate test performance characteristics by distribution of cases (defined primarily by severity of infection and time since infection/symptoms/PCR detection) but note that:

- Sensitivity and specificity ‘in our hands’ was investigated by testing 618 samples from the pre-COVID-19 era (1 marginal false positive precisely at the diagnostic threshold) and 50 samples confirmed as positive in a COVID-19 convalescent plasma study protocol (with 1 false negative).
- For epidemiological interpretation, we take seroprevalence as a close proxy of the prevalence of the condition of having been infected with SARS-CoV-2 at some point. The Elecsys Anti-SARS-CoV-2 assay appears to have particularly good durability of antibody detection for months post PCR reversion and symptom resolution, with no evidence of antibody waning and seroreversion over more than four months in a US COVID-19 convalescent plasma cohort (personal communication, M. Busch).
- We ignore, for now, the effects of 1) the donor deferral rule that people with confirmed SARS-CoV-2 infection, or COVID-19-like symptoms, are precluded from donation for a period of two weeks after PCR test and symptom resolution, and 2) deferral of regular donors who were in quarantine due to a positive contact who would not have presented for their routine donation. Given the high rate of asymptomatic infection, this is a relatively minor limitation.

We did not perform structured sampling in the sense of a primary or secondary unit of analysis, with steps of random selection of primary sampling units, households or individuals – the study merely observed donors who happen to present themselves at donation facilities on collection days. Prevalence was estimated by typical categorical and continuous predictors (age, sex, race and province) by standard methods, using the R platform for statistical computation. Although we are not aware of any biological basis for expecting racial differences – in South Africa, as elsewhere, race is, for historical reasons, a strong correlate of socio-economic status, living conditions, and social circumstances, and therefore a suspected predictor of prevalence. Provincial weighted seroprevalence estimates are based on population size estimates from Machemedze et al (3), and racial breakdown of provinces according to the 2011 census (4). The level of (dis)aggregation for headline results was chosen based on exploratory analysis described below.

## Results

During the seven collection days held throughout January 2021, 4858 voluntary non-remunerated blood donors from four provinces (EC, NC, FS and ZN) of South Africa were tested for anti-SARS-CoV-2 antibodies. The majority of blood donors were from ZN at 2107 (43%), followed by EC at 1457 (30%), FS at 831 (17%) and NC at 463 (10%).

**Figure 1:**
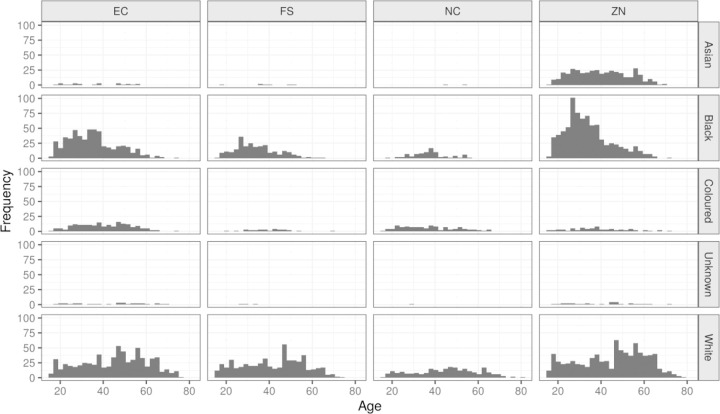
Age distribution of South African blood donors in the data set used for this analysis, decomposed by race and province (EC=Eastern Cape, FS=Free State, NC=Northern Cape, ZN=KwaZulu Natal).

There were slightly more male donors (53%). The large majority of donors in our study were White (47%) and Black (34%) with the remainder distributed relatively evenly between the Asian and ‘Coloured’ – a uniquely South African racial label indicating persons with a significant mix of ancestry from, amongst other lineages, South Asia, Indonesia, Southern Africa and Europe (5) – donors. Figure 1 shows the age distribution of donors included in the present analysis, further decomposed by race and province.

**Figure 2:**
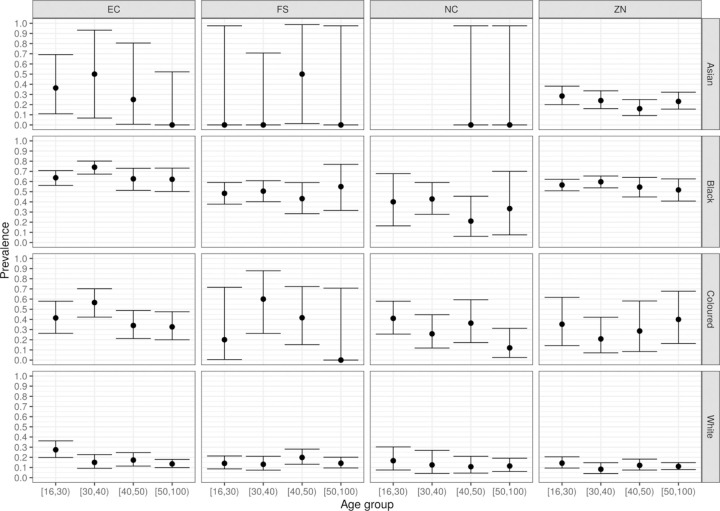
Prevalence of SARS-CoV-2 antibodies among South African blood donors, by age and race (A=Asian, B=Black, C=Coloured, W=White), for four provinces (EC=Eastern Cape, FS=Free State, NC=Northern Cape, ZN=KwaZulu Natal), expressed as a fraction between 0 (0%) and 1 (100%).

**Figure 3:**
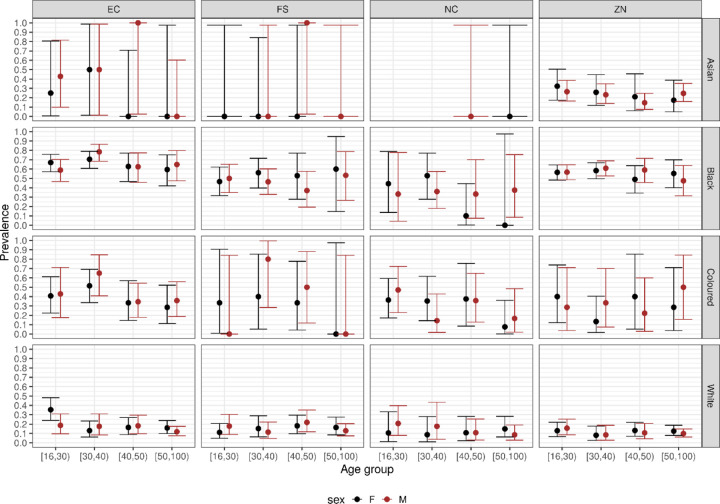
Prevalence of SARS-CoV-2 antibodies among South African blood donors, compared by sex, for each combination of i) broad age group, ii) major race group, and ii) province. (EC=Eastern Cape, FS=Free State, NC=Northern Cape, ZN=Kwazulu Natal), expressed as a fraction between 0 (0%) and 1 (100%).

After categorizing by broad age bins for four provinces and the major race groups (Figure 2), there was no association between seroprevalence and age; when sex was included (figure 3) there was no association between seroprevalence and sex. Therefore for the remaining analysis we aggregated over all ages and did not disaggregate sex.

**Figure 4:**
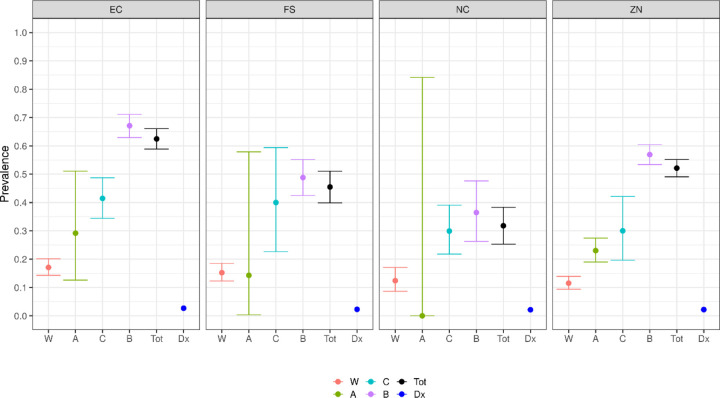
Estimated prevalence of SARS-CoV-2 antibodies, expressed as a fraction between 0 (0%) and 1 (100%), among blood donors, in January 2021, for four South African provinces (EC=Eastern Cape, NC=Northern Cape, FS=Free State, ZN=KwaZulu Natal) by primary locally used racial designations (W)hite, (A)sian, (C)oloured and (B)lack, as well as a race-weighted (Tot)al provincial estimate and the official prevalence of having had clinical Covid-19 diagnosis (Dx) by PCR of nasal or oropharyngeal swab specimens.

Figure 4 shows the seroprevalence estimates by the remaining meaningful disaggregation – race and province. The large difference by both race and province are highly statistically significant as well as epidemiologically meaningful. We also show the race-weighted overall provincial prevalence estimates (which we interpret as provincial ‘attack rates’), and the official prevalence of having been diagnosed, based on reporting of positive PCR diagnostic test results to the National Institute for Communicable Diseases (6).

Table 1 shows our provincial estimates of attack rates, as a percentage; the implied number of infections; the number of laboratory confirmed cases according to the National Institute for Communicable Diseases (NICD) (6) which reports on testing performed both in the private and public sector; and the (multiplicative) discrepancy between our estimate and the official count. Note that our estimated number of infections is conservatively based on our estimated prevalence being applied only to the age group 15–69, so these factors are not quite as large as implied by Figure 4. The estimated seroprevalence ranges from 31.8% in NC to 62.5% in the EC and is at least 8 fold higher than the official case count.

## Discussion

Our study confirmed high seroprevalence rates, in excess of 60%, among Black donors, with little sign of significant population level immunity among other race groups. These significant differences most likely can be explained by historically based socio-economic factors which hinder the implementation of COVID-19 preventative measures at a community level. Previous seroprevalence estimates from South Africa, specifically the Western Cape province (not included in our analysis), earlier in the epidemic found: 1) a very high prevalence (30–40 percent) among pregnant women attending state sector antenatal care, and people living with HIV presenting for routine viral load assessment (7); and 2) higher prevalence among workers with lower socioeconomic status (8). A review of population-based seroprevalence studies in Europe, America and Asia reported a seroprevalence range of <0.1% to more than 20% in the different regions (2).

For an indication of the meaning of such high seroprevalence values, in a one year old epidemic, consider: A prevalence of 50%, accumulated over 50 weeks, of a condition with a duration of infectiousness of 1 week, implies an average ‘prevalence of infectiousness’ of 1% of the population, with inevitable significant elevations above this average value during peaks. For people reliant on public transport, or working in public spaces, it will be difficult to limit close encounters to fewer than 100 people on any given day – i.e. difficult to encounter fewer than one infectious person per day.

We do not claim that blood donors are a perfectly representative of the South African population. Firstly, Black and White donors each account for roughly half the total participants of this study, though South Africa’s population is about 80 percent Black African and only 8 percent White/European (4). Other population groups are generally insignificantly small except Asian in Kwazulu-Natal (about 20%) and Coloured in the Western Cape (about 50%). Furthermore, repeat blood donors (who supply the majority of donations) are pre-selected to have recently been negative for pathogens included in routine blood safety screening. In South Africa this selection for being HIV negative is certainly relevant, given the country’s extraordinary HIV prevalence. Communities which are economically stressed, or without ease of access to blood donor centres, will be under-represented among the study population.

Survey dates represented in this analysis are barely past South Africa’s ‘second peak’ in COVID-19 incidence. Deferral rules based on confirmed infection or COVID-19-like symptoms should slightly depress seroprevalence estimates relative to ‘true’ prevalence. The age weighting we adopted to estimate total infections also produces a face value underestimate for population totals as it assigns no cases in the age range 0–14, which accounts for about 30 percent of the population. The Elecsys Anti-SARS-CoV-2 antibody assay appears to have particularly good detection sensitivity for months post PCR reversion (9), though there may be some seroreversion. Therefore, while further investigation of the issue of representativeness will clearly need to be done, our estimates are subject to downward bias by at least some obvious considerations.

With due consideration to both the patent and latent limitations of our study, the key observations we wish to make at this point are:

- The ordering of the prevalence-by-province and prevalence-by-race is what one would expect, based on official case counts, and an understanding of South African society.
- The particularly high attack rates in majority Black communities points to the limitations, thus far, of non-pharmaceutical interventions in the context of economic deprivation and high population density.
- The high seroprevalence amongst Black donors also raises interesting and important questions about the level of collective immunity thus far obtained through the two primary infection waves to date – but we caution against simplistic interpretations, given that substantial outbreaks have been seen in cities *after* the observation of very high seroprevalence (10), and the recent concerns about vaccine efficacy against new variants.
- The low seroprevalence amongst White donors suggests that predominantly White suburban communities lack meaningful collective immunity, and should take infection control measures very seriously for the foreseeable future.
- Given the relatively low marginal cost of leveraging the infrastructure of the blood services, we are keen to further probe the representativeness of blood-donor-based seroprevalence surveys, and to see to what extent surveillance in the blood services can be a valuable and efficient ongoing activity during major infectious disease outbreaks.

We are mindful of the incompleteness of this project, and the potential to misconstrue the wider ramifications of these findings. We endeavour to proceed rapidly from data gathering to publication, and to offer realistic and grounded interpretations of the findings.

## Figures and Tables

**Table 1:** The headline results of figure 4, in numbers: Provincial seroprevalence estimates, interpreted as attack rates, the total number of infections this implies, as well as the official number of persons diagnosed in each province, and the multiplicative factor (rounded to the nearest whole number) relating our estimate to the official case count. Note that the estimated number of infections is based on our estimated prevalence being applied only to the age group 15–69.

Province	Estimated seroprevalence / attack rate (%)	Estimated total infections (count, rounded)	Officially diagnosed cases (Count)	Diagnostic underestimate ( -fold, rounded)
Eastern Cape	62.5(58.9–66.1)	2,729,448	186,771	15
Northern Cape	31.8((25.3–38.3)	235,053	29,558	8
Free State	45.5(39.9–51.1)	878,206	70,511	12
KwaZulu Natal	52.1(49.1–55.2)	3,955,859	279,974	14

## References

[1] Goudsmit J. The paramount importance of serological surveys of SARS-CoV-2 infection and immunity. Eur J Epidemiol. 2020;35(4):331–3. Available from: 10.1007/s10654-020-00635-2 32318914 PMC7173354

[2] Lai CC, Wang JH, Hsueh PR. Population-based seroprevalence surveys of anti-SARS-CoV-2 antibody: An up-to-date review. Int J Infect Dis. 2020;101:314–22. Available from: 10.1016/j.ijid.2020.10.011 33045429 PMC7546669

[3] Machemedze T, Kerr A, Dorrington R. WIDER Working Paper 2020/67-South African population projection and household survey sample weight recalibration. 2020 [cited 2021 Feb 7]; Available from: 10.35188/UNU-WIDER/2020/824-5

[4] Total population of South Africa 2018, by ethnic groups: Statista Research Department; 11 3, 2020 Available from: https://www.statista.com/statistics/1116076/total-population-of-south-africa-by-population-group/

[5] Daya M, Der Merwe L, Galal U, Möller M, Salie M, Chimusa ER, A panel of ancestry informative markers for the complex five-way admixed South African Coloured population. PLoS One. 2013;8(12). 10.1371/journal.pone.0082224 PMC3869660 24376522

[6] NICD. COVID-19 weekly epidemiology brief. 2021 p. 1 Available from: https://www.nicd.ac.za/wp-content/uploads/2021/01/COVID-19-Weekly-Epidemiology-Brief-week-2-2021.pdf

[7] Hsiao M et al, SARS-CoV-2 seroprevalence in the cape town metropolitan sub-districts after the peak of infections. COVID-19 Special Public Health Surveillance Bulletin, 18 (S5)

[8] Shaw Higher SARS-CoV-2 Seroprevalence in Workers with Lower Socioeconomic Status in Cape Town, South Africa. Research Square, DOI: 10.21203/rs.3.rs-136543/v1 PMC7906413 33630977

[9] Busch MP. private communication (2 2021).

[10] Sabino EC, Buss LF, Carvalho MPS, Prete CA, Crispim MAE, Fraiji NA, Resurgence of COVID-19 in Manaus, Brazil, despite high seroprevalence. Lancet (London, England). 2021;397:452–5. Available from: http://www.ncbi.nlm.nih.gov/pubmed/33515491 10.1016/S0140-6736(21)00183-5 PMC7906746 33515491

